# Multiple Labeling of Compartmentalized Cortical Neurons in Microfluidic Chambers

**DOI:** 10.21769/BioProtoc.4911

**Published:** 2024-01-05

**Authors:** Guillermo Moya-Alvarado, Alejandro Aguirre-Soto, Francisca C. Bronfman

**Affiliations:** 1 Department of Physiology, Faculty of Biological Sciences and Center for Aging and Regeneration (CARE), Pontificia Universidad Catolica de Chile, Av. Libertador Bernardo O´Higgins 340, Santiago, 8970117, Chile; 2 NeuroSignaling Lab (NESLab), Institute of Biomedical Sciences (ICB), Faculty of Medicine, and Faculty of Life Sciences, Universidad Andres Bello, Echaurren 183, 8370146, Santiago, Chile

**Keywords:** Compartmentalized cultures, Microfluidic chambers, Cortical neurons, Endocytosis, BDNF, Axons, Long-distance signaling, Cholera toxin subunit B

## Abstract

Neurons are complex cells with two distinct compartments: the somatodendritic and the axonal domains. Because of their polarized morphology, it is challenging to study the differential cellular and molecular mechanisms that occur in axons and impact the soma and dendrites using conventional in vitro culture systems. Compartmentalized cultures offer a solution by physically and chemically separating the axonal from the somatodendritic domain of neurons. The microfluidic chamber model presented in this work is valuable for studying these mechanisms in primary cortical cultures derived from rat and mouse. In addition, this chamber model is compatible with various microscopy methods, such as phase contrast, and fluorescence imaging of living and fixed cells.

Key features

• Preparation and attachment of PDMS microfluidic chambers to glass coverslips.

• Primary culture of cortical neurons and plating cortical neurons in microfluidic chamber.

• Confirmation of compartmentalization using the retrograde transport of the fluorescently labeled form of cholera toxin subunit B (f-Ctb).

• Immunofluorescence and multilabeling of compartmentalized cortical neurons.

• Retrograde transport of fluorescently labeled BDNF.

## Background

The nervous system (NS) connectivity depends on the polarized morphology of neurons, as they receive multiple inputs across dendrites and integrate and then propagate responses through a single axon. This specialized morphology depends on proper neuronal intracellular transport achieved by microtubule-based molecular motors, such as dynein and kinesins. The classic view is that the flow of information in neuronal circuits is from dendrites to axons; however, there are local events in axons that are communicated to the somatodendritic domain. Local axonal processes include, among others, the local translation of proteins after injury or retrograde transport of signaling endosomes initiated by extracellular cues (Hirokawa et al., 2010; Panayotis et al., 2015; Yamashita and Kuruvilla, 2016; Guedes-Dias and Holzbaur, 2019; Pathak et al., 2021). Axonal translation has been reported for various physiological and pathophysiological processes in the peripheral NS (PNS) and in the central NS (CNS). In the PNS, axonal local translation of proteins is required for regenerative responses in cell bodies upon axonal injury (Koley et al., 2019). In the CNS, the translatome of visual circuit axons has been established (Shigeoka et al., 2016), and local protein synthesis in the presynaptic compartment has been described as a ubiquitous feature of the adult brain (Hafner et al., 2019).

The endocytic system of axons actively participates in neuronal homeostasis. In the PNS, signaling endosomes are essential for long-range signaling from the axon to the nucleus, impacting cell survival (Yamashita and Kuruvilla, 2016; Villarin et al., 2016). In the CNS, our recent findings suggest a role for signaling endosomes in the wiring of neuronal circuits (Moya-Alvarado et al., 2023). Indeed, we have demonstrated that signaling endosomes transmit retrograde signals from the axon to the nucleus in a dynein-dependent manner, increasing dendritic branching in cortical neurons. Additionally, in the hippocampus, signaling endosomes locally regulate neurotransmission in the presynaptic compartment (Andres-Alonso et al., 2019; Moya-Alvarado et al., 2022 and 2023; Lazo and Schiavo, 2023).

The cellular and molecular aspects that govern axonal homeostasis, such as those discussed above, and how they impact the somatodendritic domain are difficult to study in normal in vitro cultures because we are unable to control the microenvironment of the axonal compartment. There are different types of compartmentalized systems for the in vitro culturing of neurons, including microfabricated platforms (Campenot, 1977), scaffold-based systems (Zhang et al., 2022), and microfluidic chambers (Park et al., 2006). Microfabricated platforms, such as Campenot chambers, were first developed to study the role of trophic factors applied to long-projecting neurons of the PNS (MacInnis and Campenot, 2002). However, the utility of Campenot chambers has remained limited given the arduous manufacturing process, incompatibility with high-resolution optical imaging, and limited throughput (Jadhav et al., 2016). In this protocol, we describe the use of a microfluidic chamber fabricated using lithography in polydimethylsiloxane (PDMS) as a platform for culturing primary cortical neurons from rat or mouse embryos. The advantages of microfluidic systems include compatibility with different types of neurons, improved fluidic compartmentalization, and enhanced imaging capabilities (Southam et al., 2013; Stuardo et al., 2020; De Vitis et al., 2021). Here, we describe a protocol to prepare and assemble microfluidic chambers from epoxy molds and to use them as a platform for primary culture of cortical neurons for fluorescence microscopy analysis of retrogradely transported proteins.

## Materials and reagents

1. Mice (*Mus musculus*) or rats (*Rattus norvegicus*) embryos (16–18 days old)
2. Hank’s Balanced Salt Solution plus calcium and magnesium (HBSS) (Gibco, catalog number: 14025134)
3. Modified Eagle’s medium (MEM), high glucose, pyruvate (Gibco, catalog number: 11995081)
4. Horse serum (Gibco, catalog number: 16050122)
5. Coverslips (Marienfeld, catalog number: 0111650)
6. Neurobasal medium (Gibco, catalog number: 21103049)
7. B27 (Gibco, catalog number: 17504044)
8. GlutaMax supplement (Gibco, catalog number: 35-050-061)
9. Penicillin/streptomycin (10,000 U/mL) (Gibco, catalog number: 15140-122)
10. Cytosine B-D-arabinofuranoside (AraC) (Sigma-Aldrich, catalog number: C1768)
11. Microfluidic chamber (Xona microfluidics, catalog number: RD450 or SND450) or epoxy microfluidics molds. The molds used to prepare the compartmentalized chambers were fabricated at the microfluidic core facility of Tel Aviv University and donated by Professor Eran Perlson from the Department of Physiology and Pharmacology, Sackler Faculty of Medicine, Tel Aviv University (Gluska et al., 2016)
12. Cholera toxin subunit B (recombinant), Alexa Fluor 555 (f-Ctb555) (Invitrogen, catalog number: C34776)
13. Cholera toxin subunit B (recombinant), Alexa Fluor 647 (f-Ctb647) (Invitrogen, catalog number: C34778)
14. Mouse anti-βIII tubulin (Sigma, catalog number: T8578)
15. Rabbit anti-Rab11a (Invitrogen, catalog number: 715300)
16. Donkey anti-mouse IgG, Alexa 647 (Invitrogen, catalog number: A31571)
17. Donkey anti-rabbit IgG, Alexa 488 (Invitrogen, catalog number: A21206)
18. Poly-D-lysine (PDL) (Corning, catalog number: 354210)
19. Natural mouse laminin (Gibco, catalog number: 23017015)
20. Antibiotic/antimycotic 100× (Gibco, catalog number: 15240062)
21. 6-well plate (Corning, catalog number: 15240062)
22. Cell culture dish 35 mm (Corning, catalog number: 353001)
23. Sylgard^TM^ 184 silicone elastomer kit (Dow Corning, catalog number: 4019862)
24. Trypsin 2.5% 10× (Gibco, catalog number: 15090046)
25. Mowiol 4-88 (Millipore, catalog number 475904)
26. Detergent Micro90 cleaning solution (Cole-Parmer, catalog number: 18100-05)
27. Ethanol (TCL Group, catalog number: IN-0150)
28. Distilled water (Sanderson, catalog number: SAN0003)
29. Phosphate buffered saline (PBS) 10× (Winkler, catalog number: BM-1340)
30. Glucose (Merck, catalog number: 108337)
31. Boric acid (Sigma-Aldrich, catalog number: B6768)
32. Borax anhydrous (Sigma-Aldrich, catalog number: B0127)
33. Syringe driven filters 0.22 µm (Jet Biofil, catalog number: JET-022)
34. Triton X-100 (Merck, catalog number: K42092903 220)
35. Hoechst (Invitrogen, catalog number: R37605)
36. Paraformaldehyde (Sigma, catalog number: 158127)
37. Sucrose (Merck, catalog number: 1.07687.1000)
38. Biopsy punch (Miltex, catalog number: 33-55)
39. Single edge blade (Stanley, catalog number: 28-510)
40. Cell culture dish 150 mm × 25 mm (SPL Life Sciences, catalog number: 201505SPL)
41. Parafilm (Amcor, catalog number: PM-996)
42. 15 mL conical tubes (SPL Life Sciences, catalog number: 50015)
43. 50 mL conical tubes (SPL Life Sciences, catalog number: 50150)
44. Pasteur pipettes (SPL Life Sciences, catalog number: 91010)
45. HEPES (Sigma, catalog number: 1001889449)
46. Sodium bicarbonate (Merck, catalog number, K50321729 832)
47. Bovine serum albumin (BSA) (Jackson ImmunoResearch Laboratories, catalog number: 001-000-162)
48. TrkB-fc (B&D Systems, catalog number: 688TK)
49. Fish gelatin (Sigma, catalog number: G7765)
50. Saponin (Sigma, catalog number: S4521)
51. Human BDNF-biotin (Alomone labs, catalog number: B-250-B)
52. Transferrin from human serum, Alexa 555 conjugate (Invitrogen, catalog number: T35352)
53. Streptavidin DyLight 488 (Invitrogen, catalog number: 21832)

**Solutions**

1. Polydimethylsiloxane (PDMS) mixture (see Recipes)
2. Poly-D-lysine (PDL) with laminin (see Recipes)
3. Borate buffer pH 8.3 (see Recipes)
4. HBSS 1× (see Recipes)
5. HBSS-trypsin (see Recipes)
6. MEM-HS (100 mL) (see Recipes)
7. Neurobasal-B27 (see Recipes)
8. Neurobasal-B27 with AraC (see Recipes)
9. Neurobasal-Cholera toxin subunit B (see Recipes)
10. Paraformaldehyde with sucrose (PFA solution) (see Recipes)
11. Blocking solution (see Recipes)
12. Antibody solution (see Recipes)

**Recipes**

1. **Polydimethylsiloxane (PDMS) mixture**
  **Table:** 

Reagent	Final concentration	Quantity
PDMS base (part A)	n/a	40.5 g
PDMS curing (part B)	n/a	4.5 g
Total		45 g
*Note: Sylgard^TM^ 184 silicone elastomer kit contains the two different PDMS viscous solutions.*
2. **Poly-D-lysine (PDL) with laminin**
  **Table:** 

Reagent	Final concentration	Quantity
Poly-D-lysine (1 mg/mL) prepared in borate buffer pH 8.3	0.1 mg/mL	1 mL
Laminin	20 µg/mL	0.1 mL
H_2_O	n/a	8,900 mL
Total		10 mL
3. **Borate buffer pH 8.3**
  **Table:** 

Reagent	Final concentration	Quantity
Boric acid	3.1 mg/mL	155 mg
Borax anhydrous	4.75 mg/mL	237.5 mg
H_2_O	n/a	50 mL
Total		50 mL
*Note: Mix boric acid and borax anhydrous in distilled water using a magnetic bar and mix the solution overnight with a magnetic stirrer. After, adjust the pH and filter through a syringe driven filter (0.2 µm).*
4. **HBSS 1×**
  **Table:** 

Reagent	Final concentration	Quantity
HBSS 10×	1×	10 mL
HEPES pH 7.4 1 M	10 mM	1 mL
Sodium bicarbonate	7.5%	0.5 mL
Antibiotic/antimycotic 100×	1×	1 mL
Autoclaved H_2_O	n/a	87.5 mL
Total		100 mL
5. **HBSS-trypsin**
  **Table:** 

Reagent	Final concentration	Quantity
10× Trypsin	1×	1 mL
HBSS 1×	1×	9 mL
Total		10 mL
6. **MEM-HS (100 mL)**
  **Table:** 

Reagent	Final concentration	Quantity
Horse serum	10%	10 mL
Glucose	0.6%	3 mL
GlutaMax 100×	1×	1 mL
Antibiotic/antimycotic 100×	1×	1 mL
MEM 1×	1×	86 mL
Total		100 mL
7. **Neurobasal-B27**
  **Table:** 

Reagent	Final concentration	Quantity
B27	2%	2 mL
GlutaMax 100×	1×	1 mL
Penicillin/streptomycin 100×	1×	1 mL
Neurobasal	1×	96 mL
Total		100 mL
8. **Neurobasal-B27 with AraC**
  **Table:** 

Reagent	Final concentration	Quantity
AraC 1 mM	1 µm	10 µL
Neurobasal-B27 (Recipe 7)	n/a	990 µL
Total		1 mL
9. **Neurobasal-Cholera toxin subunit B**
  **Table:** 

Reagent	Final concentration	Quantity
Cholera toxin subunit B Alexa 1 mg/mL	1 μg/mL	1 µL
Neurobasal	n/a	999 µL
Total		1 mL
10. **Paraformaldehyde with sucrose (PFA solution)**
  Warm up PBS to 65 °C and add paraformaldehyde and sucrose. Mix every 5 min to completely dissolve the paraformaldehyde and complete volume to 5 mL with PBS 1×.
  **Table:** 

Reagent	Final concentration	Quantity
PBS 1×	n/a	Approximately 5 mL
Paraformaldehyde	4%	0.2 g
Sucrose	4%	0.2 g
Total		5 mL
11. **Blocking solution**
  **Table:** 

Reagent	Final concentration	Quantity
BSA	3%	0.3 g
Fish gelatin	5%	50 µL
Saponin 10% in PBS	0.2%	20 µL
PBS 1×	n/a	930 µL
Total		1 mL
12. **Antibody solution**
  **Table:** 

Reagent	Final concentration	Quantity
BSA	3%	0.3 g
Fish gelatin	5%	50 µL
Saponin 10% in PBS	0.02%	2 µL
PBS 1×	n/a	948 µL
Total		1 mL

**Laboratory supplies**

1. Pipette p2 (Thermo Scientific Finnpipette^®^ F2, catalog number: 4642010)
2. Pipette p10 (Thermo Scientific Finnpipette^®^ F2, catalog number: 4642040)
3. Pipette p20 (Thermo Scientific Finnpipette^®^ F2, catalog number: 4642060)
4. Pipette p200 (Thermo Scientific Finnpipette^®^ F2, catalog number: 4642080)
5. Pipette p1000 (Thermo Scientific Finnpipette^®^ F2, catalog number: 4642090)
6. Alcohol burner (Usbeck), butane/propane mix (Providus s.r.l. 75% butane, 25% propane)
7. Neubauer counting chamber (HGB Germany)
8. Glass vacuum desiccator 210 mm (ISOLAB GmbHha, see Figure 1A)
9. HYBAID Incubator Shake 'n' Stack (Thermo Scientific, catalog number: HBMOVCST220)
10. Mini dialysis devices, Slide-A-Lyzer (Thermo Fisher, catalog number: 69590)

## Equipment

1. Forceps short and extra fine length 110 mm (Dumont, catalog number: 11251-20)
2. Fine scissors 11.5 cm, 4 1/2" (Rudolf, catalog number: RU 1631-11 M)
3. Stereomicroscope for dissection (Leica, model: S6D L2)
4. Inverted phase contrast microscopy to visualize cells (Brand Motic, model: AE31E)
5. Horizontal laminar flow hood (Labtech, catalog number: LCB-0122H)
6. Vertical laminar flow hood (Labconco, catalog number: 3620924)
7. Cell vertical incubator (Forma Scientific, model: 3111)
8. Centrifuge (Hermle Labortechnik GmbH, catalog number: Z 233 MK-2)
9. Light microscope (Motic, model: AE31E Trinocular)
10. Confocal microscope (Leica, model: TCS SP8)

## Software and datasets

1. LAS X version 3.5.5.19976 software
2. ImageJ (version 1.54J)

## Procedure

- **Preparation and attachment of PDMS microfluidic chambers to glass coverslips**
  1. Preparation of microfluidic chambers
    - Take the epoxy molds, which are in a 10 cm plastic Petri dish, and use pressurized air/N2 to blow any remaining dust off the epoxy molds (Gluska et al., 2016) (see Figure 1).
      **Figure 1.**
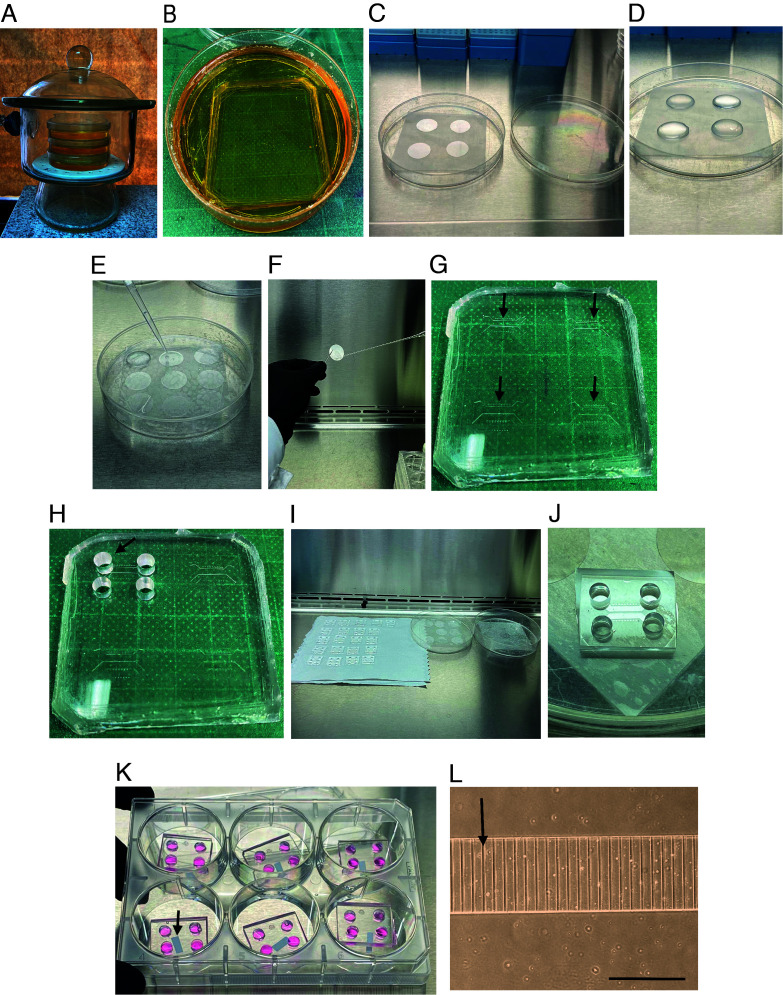
Preparing coverslips and microfluidic chambers for seeding cortical neurons. (A) Vacuum desiccator containing microfluidics molds. The molds are placed into a 10 cm plastic Petri dish as shown in B. (B) Mold before being put into the oven. (C) No. 1 glass coverslips (25 mm) placed in a piece of parafilm in a plastic dish inside the hood. (D) Each coverslip was covered with 500 µL of poly-D-lysine (PDL) and laminin mixture and incubated overnight in the cell incubator at 37 °C. (E) Coverslips are washed three times with 600 µL of autoclaved distilled water using a p1000 pipette. (F) Photograph shows how to remove the coverslip with fine forceps to dry out the water if liquid is below the cover. (G) The silicone device contains four chambers (indicated by the black arrows) that will be cut after punching the four holes. (H) Silicone device with four punch holes in the first chamber. (I) Picture shows how to place the microfluidic chambers and the coverslips in the hood for drying. (J) The microfluidic chamber is attached to the coverslips. (K) The microfluidic chambers are attached to the coverslip in a 6-well plate containing plating media. The arrow indicates a small piece of paper below the cover that was added so the cover will not stick to the plastic well. (L) Close view of the microgrooves of the microfluidic chamber with plating media before plating cells. The arrow indicates a microgroove. Scale bar, 400 µm.
    - Keep the mold plates closed until casting using a piece of parafilm around the plate.
    - Prepare the PDMS mixture at a 1:10 ratio (see Recipe 1).
    - Pour the PDMS mixture into the molds. Place the molds in a vacuum desiccator for 2–3 h until the PDMS becomes clear without air bubbles (Figure 1B).
    - Move molds into a 70 °C oven for at least 3 h, up to overnight.
    - Using gloves, carefully position a scalpel at the edge of the mold and the PDMS cast. Precisely incise along the border of the cast with the scalpel to facilitate the detachment of the PDMS from the epoxy mold. Subsequently, with the aid of a spatula, extract the PDMS cast from the mold. Then, place the PDMS cast on a flat surface to separate the chambers and punch out the holes for plating the cells.
    - Use 5 mm biopsy punchers to cut holes in the PDMS. Punch four holes to allow the flux of media from side-to-side of the channel (see Figure 1H and Figure 2).
*Note: The microgrooves must face up during this step. This will prevent disrupting microgrooves with the punch.*
      **Figure 2.**
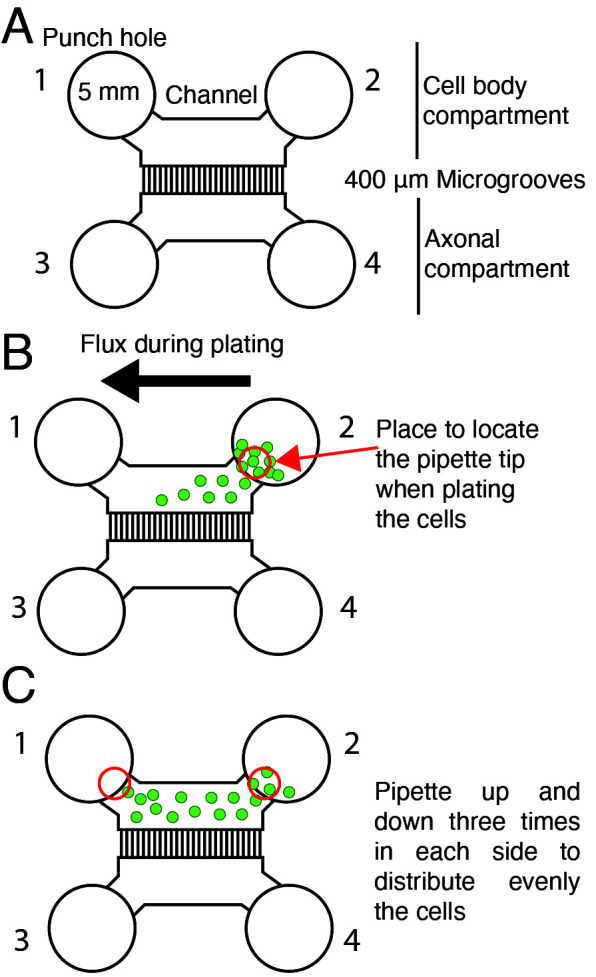
Plating cortical neurons in a microfluidic chamber. (A) Scheme of the microfluidic chamber used in this protocol. Five-millimeter punch holes are made on each side of the compartments in the chamber to allow the flux of media. Punch holes 1 and 2 define the cell body compartment; punch holes 3 and 4 define the axonal compartment. (B) When plating cells, the pipette tip is placed near the channel's entrance (red circle) and pipetted up and down in hole 2. Green dots represent cells. The black arrow shows the movement of the cells when plating. (C) Schematic representation of neuron distribution inside the cell body compartment. Red circles indicate where the pipette tip must be placed to pipette up and down and distribute the cells.
    - If the mold has more than one chamber, each chamber is cut into single chambers using a single-edge blade.
    - If you do not have the molds, you can use the commercial microfluidic chambers.
    - Place the chambers in a 10 cm plastic Petri dish and cover and seal them with parafilm until needed.
  2. Glass coverslips and chambers preparation prior to neuronal seeding (Figure 1)
    - Inside the laminar flow hood, add an 8 cm^2 ^parafilm layer into a sterile 150 mm ×25 mm plastic culture dish (Figure 1C).
    - Place the 25 mm No. 1 glass coverslips on parafilm (Figure 1C).
    - Coat each coverslip with 500 µL of PDL and laminin mixture (see Recipe 2) and incubate overnight in the incubator (Figure 1D).
    - The next day, hand wash (using plastic gloves) the microfluidic chambers for 5 min with 500 mL of 2% Micro90 detergent in distilled water. You can use your fingers to remove any dust or dirt from the chamber.
    - Place the chambers in a beaker and add 400 mL of distilled water, mix gently with a plastic spoon to remove the detergent thoroughly, and wait 10 min; repeat this process six times.
    - Put the chambers in 100 mL of 100% ethanol for 10 min at room temperature.
  3. Mounting the chamber on top of the cover
    - Remove the plastic dishes containing coverslips from the incubator and place them inside a laminar flow hood.
    - Remove the mixture of PDL and laminin with the p1000 pipette.
    - Wash the coverslips three times with 600 µL of autoclaved distilled water (using the p1000 pipette, Figure 1E).
    - Dry out the coverslips using a vacuum and wait until the cover is completely dry. Place the coverslips inside a cell culture dish with the substrate side facing up.
*Note: If there is liquid below the cover, take the cover with fine forceps and dry out the water (Figure 1F).*
    - Put the microfluidic chambers inside the hood on delicate paper wipers with the microgrooves looking up (Figure 1I).
    - Turn on the UV light from the hood and irradiate and sterilize the chambers and the coverslips for 5 min.
    - Attach the microfluidic chamber to the coverslips. Adjust the chamber to the edge of the coverslip and drop the chamber. Gently touch the chamber to seal the chamber to the coverslip (Figure 1J).
*Note: PDMS microfluidics exhibit a remarkable capacity to achieve a robust and reproducible seal with glass covers without requiring plasma-bonding procedures. This approach facilitates the removal and reusability of the microfluidic chamber for future experiments.*
    - Place the microfluidic chamber attached to the glass coverslips inside a 35 mm dish or 6-well plate (Figure 1K).
*Note: Below the glass coverslip, add a small piece of tissue paper. This will prevent the glass from adhering to the plastic (Figure 1K).*
    - Add 400 µL of MEM-HS medium (see Recipe 6) to the chamber and put it inside the incubator until use (Figure 1K).
*Notes:*
*i. At this point, you can check if the chambers are leaking by adding 200 µL of MEM-HS to the cell body compartment and wait for 2–3 min. Subsequently, observe the axonal compartment (Figure 1K) to detect any potential leaks.*
*ii. Since there is no hydrophobic bonding between the cover and the chamber, it is recommended to add MEM-HS (200 µL) directly to the channels of the cell body compartment (and not in the punch hole space) (see Figure 2A). If bubbles are left in the cell body compartment channel, you should pipette out the media with a p200 and refill until no bubbles are left.*
- **Primary culture of cortical neurons and plating neurons in microfluidic chambers**
  1. Primary cultures of cortical neurons modified from Kaech and Banker (2006) for cortical neurons (Moya-Alvarado et al., 2023)
    - Add each embryo in 10 mL of HBSS (see Recipe 4) inside a 10 cm plate on a bucket with ice.
    - Decapitate the embryos and place the heads on a 10 cm plate with 10 mL of HBSS on ice.
    - Hold the heads through the eyes and open the skin and skull with scissors to expose the dorsal side of the brain.
    - Remove the brain from the skull and place on a 10 cm plate with 10 mL of HBSS on ice.
    - Under the dissection scope, separate the brain hemispheres and remove the meninges.
    - Dissect the cortex, cut it into small pieces (0.5–1 mm^2^), and add them to a 60 mm cell culture dish containing 3 mL of HBSS 1× on ice.
    - Transfer the small pieces of cortex with a Pasteur pipette to a 15 mL conical tube with 10 mL of HBSS-trypsin solution (see Recipe 5) to digest the tissue.
    - Incubate the small pieces of cortex for 10 min at 37 °C in the incubator or water bath.
    - Prepare three Pasteur pipettes with tips of three different diameters (1, 0.75, and 0.35 mm approximately). This is achieved by placing the fine part of the pipette close to the flame of a small gas burner.
    - Wash the small pieces of cortex placed in the 15 mL conical tube three times with 5 mL of cold HBSS 1× and wait until the tissue decants in the tube.
    - Remove the HBSS 1× and resuspend the cortex in 2 mL of MEM-HS with a p1000 pipette.
    - Then, dissociate the small pieces of cortex until the solution becomes cloudy by pipetting repeatedly with glass pipettes. First, use a pipette with a larger diameter tip, and then use a pipette with a smaller diameter tip.
*Note: Pipette up and down the MEM-HS with each glass pipette before using them with the tissue. This mitigates the adhesion of tissue fragments to the glass surfaces, ensuring optimal handling of the sample.*
  2. Plating neurons in the microfluidic chambers
    - Count the cells in a Neubauer counting chamber.
    - Dilute or concentrate the cells in MEM-HS to the desired volume, depending on the number of chambers, by using centrifugation. Five microliters of MEM-HS containing 40 × 10^3^–50 × 10^3^ cells was used per chamber.
*Note: If you plate 10 chambers, you should take 50 × 10^4 ^cells (50 × 10^3 ^per chamber) and resuspend them in 50 µL of MEM-HS.*
    - Take the microfluidic chambers from the incubator and remove the MEM-HS from all four-punch holes (there will still be media in the channel compartment).
    - Add 5 µL (for a right-handed person use hole 2) of MEM-HS containing 50 × 10^3 ^cells into the chamber and pipette up and down from both sides of the cell body compartment (three times on each side) (Figure 2B and C).
*Note: To obtain better results, use a p10 tip to plate the cells inside the chamber. This approach ensures precise and consistent cell distribution, contributing to subsequent reproducible experimental results. After adding the cells, pipette up and down in both sides of the cell body compartment to allow a homogeneous distribution of cells inside the chamber.*
    - Allow the neurons to attach to the glass coverslips for 30 min inside the incubator.
    - Then, add 60 µL of MEM-HS in each punch hole (240 µL total in the four of them, as shown in Figure 2A) to fill the chamber.
    - On the next day, remove the MEM-HS from the chamber and add 80 µL of Neurobasal-B27 with AraC (see Recipe 8) in each punch hole of the cell body compartment and 40 µL of Neurobasal-B27 with AraC each punch hole of the axonal compartment (240 µL total) (see Figure 2).
*Note: To facilitate the growth of axons toward the distal axon compartment, add a total of 160 µL of Neurobasal-B27 to the cell body compartment (80 µL in hole 1 and 80 µL in hole 2, as shown in Figure 2); 80 µL in total should be added to the distal axonal compartment (40 µL in hole 3 and 40 µL in hole 4, as shown in Figure 2). To minimize cell disruption, carefully add the media directly to the wall of the punched-out holes of the microfluidic chambers. Generally, consider adding 2/3 of the total volume to the cell body compartment and 1/3 to the distal axonal compartment. The differential volume between both compartments must be at least 80–100 µL to ensure microfluidic isolation of the two compartments. This differential volume must be conserved along all the cultures if fluidic isolation is required; therefore, media supplementation must be performed every two days due to media evaporation.*
    - Change 20% of Neurobasal-B27 media every two days for a better growth of neurons at 5 days in vitro (DIV). Several axons are already grown in the axonal compartment (Figure 3).
      **Figure 3.**
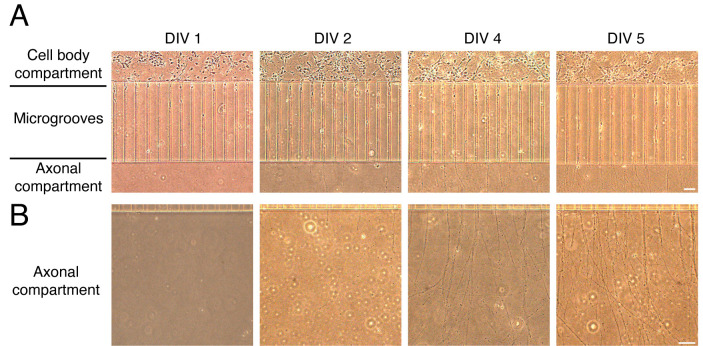
Growth of cortical neurons in microfluidic chambers. (A) Representative images of cortical neurons grown in microfluidic chambers during the days in vitro (DIV). Cortical neurons are plated in the cell body compartment. During the days in vitro, neurons extend their axons along the microgrooves. Neurons reach the axonal compartment approximately at DIV 4–5. (B) Representative images of axons during different DIV (1–5). Scale bar, 50 µm.
- **Retrograde transport of fluorescently labeled f-Ctb in compartmentalized cortical neurons**
  1. Confirmation of microfluidic chamber compartmentalization and retrograde labeling of neuronal somas
    - Ctb is a protein that is endocytosed in neurons by binding the ganglioside G_M1_ in the plasma membrane. Axonal endocytosed Ctb is efficiently retrogradely transported in neurons and accumulates in the Golgi apparatus in the cell bodies of neuronal cells in vitro and in vivo (Wang et al., 2016).
    - To study the accumulation of Ctb in neuronal cell bodies, treat 5–7 DIV compartmentalized cultures with f-Ctb in the axonal compartment overnight. At the desired DIV, remove all the media from the chamber and add 80 µL of warm Neurobasal-B27 in hole 1 and 2 (Figure 2) and 40 µL of warm Neurobasal-B27 with f-Ctb674 (1 µg/mL) (see Recipe 9) in hole 3 and 4 in the axonal compartment (Figure 2). Subsequently, transfer the cells to the incubator overnight. This procedure allows us to recognize chambers that are well compartmentalized (not all cell bodies should be labeled with f-Ctb) and to label neurons with axons in the axonal compartment (Figure 4). Therefore, if a treatment is added to the axonal compartment, e.g., BDNF, only neurons accumulating f-Ctb are considered to quantify responses. For instance, neurons presenting activation of the transcription factor cAMP response element binding protein (CREB) are responsive to BDNF, as was done in the study by Moya-Alvarado et al. (2023).
    - On the next day, wash the f-Ctb647 with neurobasal medium and add 80 µL of f-Ctb555 (1 µg/mL) to the axonal compartment for 30, 90, 120, and 180 min. These treatments are useful for visualizing the co-internalization of any fluorescently labeled protein with f-Ctb (Figure 4 and Figure 5).
      **Figure 4.**
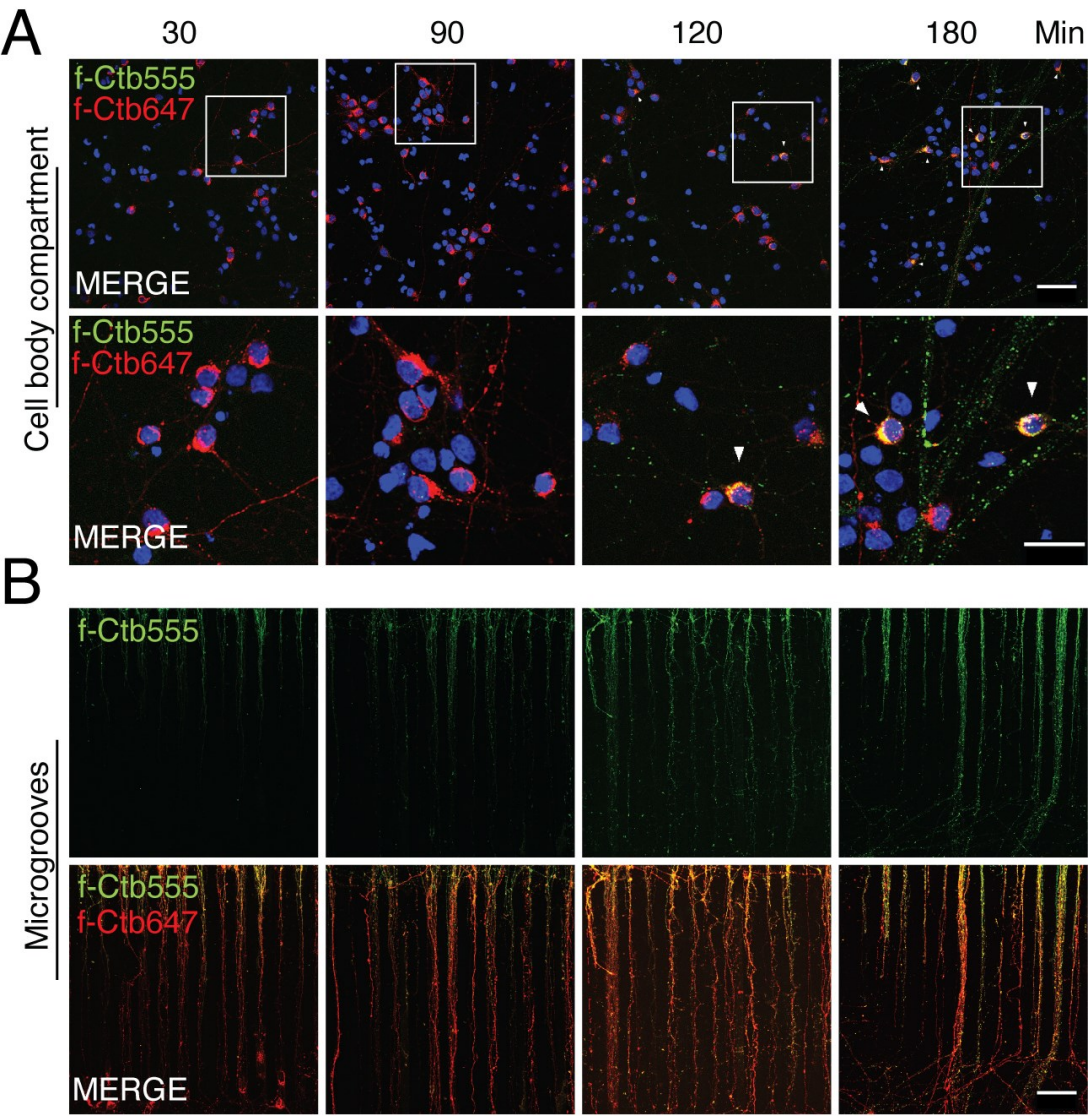
Retrograde transport of fluorescently labeled Alexa Fluor 647 and 555 cholera toxin subunit B (f-Ctb) in microfluidic chambers. Five days in vitro (DIV) cortical neurons were treated with f-Ctb647 (red) overnight. This treatment allows to verify chamber compartmentalization and label neurons that projected their axons to the axonal compartment. At 6 DIV, neurons were treated with f-Ctb555 for 30, 90, 120, and 180 min (green). Hoechst staining is shown in blue. This treatment allows to study the kinetics of f-Ctb transport and is useful for co-transport studies, as shown in Figure 5. (A) Upper panels show the cell body compartment. Scale bar, 50 µm. Lower panels show a magnified view of a group of cells shown in the square in the upper panel. Scale bar, 25 µm. White arrowheads show co-localization of f-Ctb555 and f-Ctb647. (B) Upper panels show axons in microgrooves labeled with f-Ctb555 (green). Lower panels show axons in microgrooves labeled with f-Ctb647 and f-Ctb555. Scale bar, 50 µm.
      **Figure 5.**
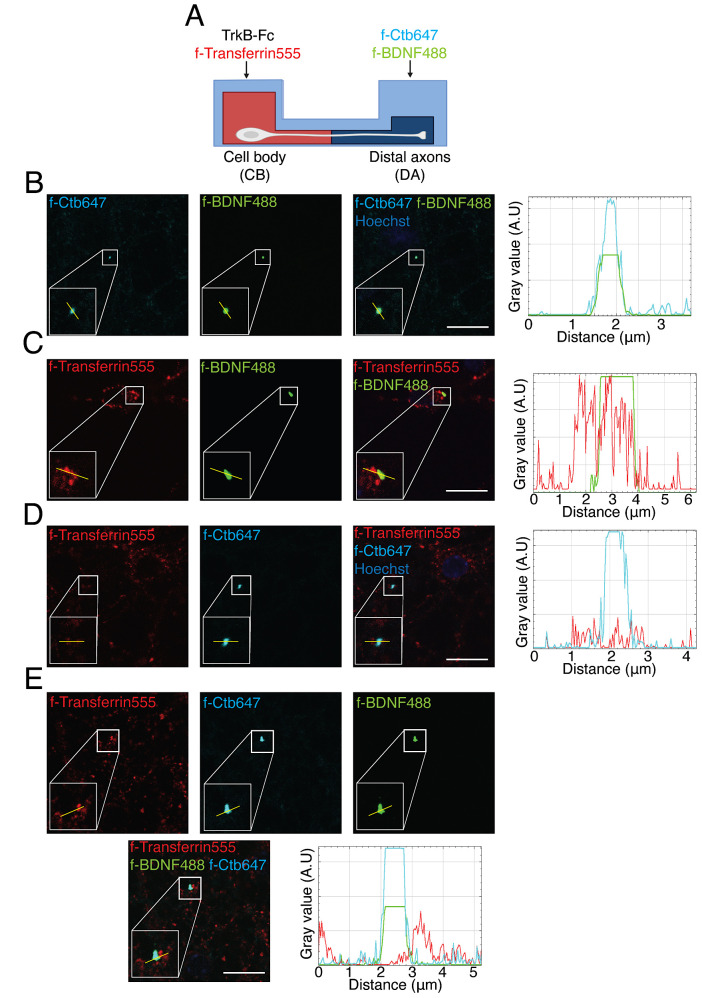
Axonal BDNF-positive endosomes co-localized with Ctb and partially with transferrin in the neuronal soma in compartmentalized cultures of cortical neurons. (A) Experimental design used to study transferrin co-localization with BDNF and Ctb retrogradely transported into neuronal cell bodies. In the cell body (CB) compartment, TrkB-Fc (100 ng/mL) was added for 60 min; then, in the axonal compartment, f-BDNF488 was added together with f-Ctb647 (1 µg/mL) for 3 h, while in the cell body compartment (CB) f-Transferrin 555 (100 µg/mL) was added to label recycling endosomes 30 min before withdrawing the treatment with f-BDNF488. (B) Images show co-localization of BDNF (green) and f-Ctb647 (cyan). The right panel shows a plot profile with the fluorescence intensity for BDNF (green) and f-Ctb647 (cyan) of the line shown in the figure. (C) Images show partial co-localization of BDNF (green) and transferrin (red). The right panel shows a plot profile with the fluorescence intensity for BDNF (green) and transferrin (red) of the line shown in the figure. (D) Images show partial co-localization of f-Ctb647 (cyan) and f-transferrin 555 (red). The right panel shows a plot profile with the fluorescence intensity for f-Ctb647 (cyan) and f-transferrin 555 (red) of the line shown in the figure. (E) Images show partial co-localization of BDNF (green), f-Ctb647 (cyan), and f-transferrin 555 (red). In the lower right panel, a plot profile with the fluorescence intensity for f-BDNF488 (green), f-Ctb647 (cyan), and f-transferrin 555 (red) from the line shown in the figure is shown. These are representative images of an experiment and were acquired using the UNAB Leica SP8 microscope at 63× magnification with a 5× digital zoom. Scale bar, 10 µm.
    - After the incubation, remove the media from the microfluidic chambers with a p200 pipette.
    - Wash the chambers with 200 µL of PBS one time on each side of the chamber (cell body and axonal compartment).
    - Carefully remove the chamber from the side of the coverslip to re-use the microfluidic chamber for another experiment before fixing the neurons.
*Note: This procedure can be pursued either within the plate where the cells were initially cultured or by transferring the glass coverslips, bearing the neurons facing upward, over a piece of parafilm placed inside a container with a wet paper towel to maintain humidity (humid chamber). Additionally, it is possible to fix the neurons while the microfluidic chamber is still attached, but this is not recommended if the intention is to re-use the chamber afterward.*
    - To fix the cells, add 100 µL of PFA solution (see Recipe 10) to the neurons and incubate them for 18 min at room temperature.
*Note: To avoid detachment of neurons from the coverslips, add the solution slowly through the edge of the glass coverslip.*
    - Remove the PFA solution and carefully wash three times with 100 µL of PBS for 5 min each wash.
    - Incubate the samples with 100 µL of Hoechst staining solution (1:5,000) for 10 min.
    - Wash the cells with 100 µL of PBS three times for 3 min each and then one time with 100 µL of distilled water to remove the salts from the PBS. Carefully drop the coverslip into 50 µL of mounting media (Mowiol 4-88) with the neurons facing the slide.
    - Dry the slides at room temperature for at least 12 h and then store the samples at 4 °C until imaging. Drying time may vary with different types of mounting media.
*Note: To identify neurons that have projected their axons into the axonal compartment, the Ctb tracer must undergo an incubation period of a minimum of 3 h within the axonal compartment. This temporal requirement is crucial for optimal uptake and retrograde transport of Ctb, ensuring reliable neuronal labeling for subsequent analysis.*
- **Immunofluorescence and multiple labeling of compartmentalized cortical neurons**
  1. Multiple labeling of f-Ctb, endocytic structures, and βIII-tubulin identifying microtubules in axons and cell bodies
    - At 5–7 DIV, remove the media from the chamber and add a total of 240 µL of warm Neurobasal-B27: 160 µL to the cell body compartment (80 µL in hole 1 and 2) and 80 µL with f-Ctb555 (1 µg/mL) in the axonal compartment (40 µL in hole 3 and 4). Incubate for at least 3 h in the incubator (Figure 2).
    - Remove the media from the four holes of the microfluidic chambers with a p200 pipette.
    - Perform three quick washes of the neurons with 50 µL of PBS (at room temperature) in each of the four punch holes of the chamber, as shown in Figure 2.
    - Carefully remove the chamber from the side of the coverslip to re-use the microfluidic chamber for another experiment.
*Note: We re-used microfluidic chambers 2–3 times. Chambers are re-washed as indicated in Section A, step 2d and 2e.*
    - Gently add 100 µL of PFA solution (at room temperature) to the neurons and incubate them for 18 min at room temperature.
    - Remove the PFA solution and carefully wash three times with 100 µL of PBS for 5 min each wash.
    - Remove PBS and add 150 µL of blocking solution (see Recipe 11) for 1 h at room temperature.
*Note: For the staining described here, we used BSA and fish gelatin combined with saponin as a cell permeabilizer to block the samples. In our hands, this blocking buffer allows better visualization of endosomes in neuronal cells in vitro. If you wish to stain nuclear proteins, you will have to replace the saponin for Triton X-100 (see Moya-Alvarado et al., 2023). Additionally, BSA can be replaced by serum in the blocking solution that matches the species of the secondary antibody to decrease nonspecific binding.*
    - Remove the blocking solution and add 100 µL of primary antibody solution (see Recipe 12). Then, incubate the cells with mouse anti-βIII tubulin (1:750) and rabbit anti-Rab11a (1:400).
*Note: The concentration, condition, and time of incubation of the primary and secondary antibodies can vary depending on the antibody used during the experiment.*
    - Incubate the neurons with primary antibody solution overnight at 4 °C.
    - Then, wash the primary antibody three times with 100 µL of PBS for 5 min each.
    - Remove the PBS and incubate the neurons with 100 µL of secondary antibody dissolved in antibody solution for 1 h at room temperature. For the staining illustrated in this protocol, we used donkey anti-mouse Alexa 647 (1:500) and donkey anti-rabbit Alexa 488 (1:500).
    - Wash the secondary antibody three times with 100 µL of PBS for 5 min each.
    - Incubate with 100 µL of Hoechst solution (1:5,000) for 10 min.
    - Wash the cells with 100 µL of PBS three times for 3 min each and then one time with 100 µL of distilled water to remove the salts from the PBS. Put the coverslip in a slide with mounting media (Mowiol 4-88).
    - Dry the slides at room temperature for at least 12 h and then store the samples at 4 °C until imaging. Representative images of this protocol are shown in Figure 6.
      **Figure 6.**
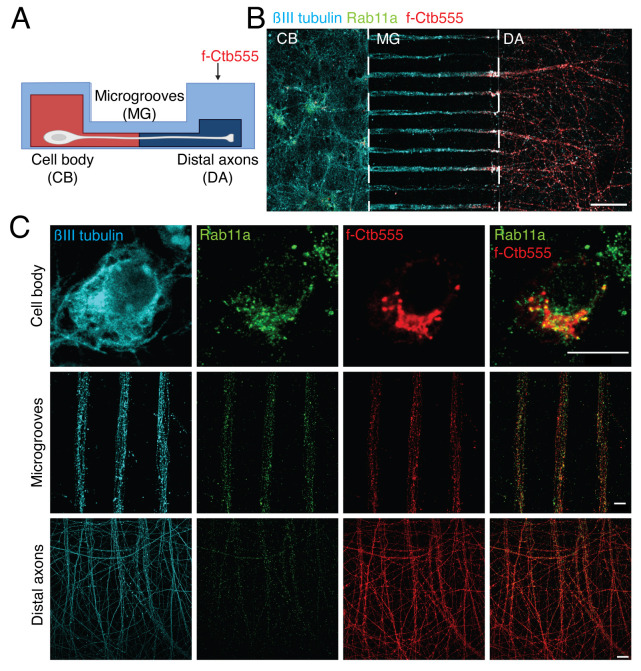
Multiple labeling of cortical neurons in microfluidic devices. (A) Scheme of the experimental design to label the neurons in microfluidic chambers. Five days in vitro (DIV) cortical neurons were treated with f-Ctb647 (red) overnight to stain all the neurons that projected their axons. At 6 DIV, neurons were fixed and immunostained. (B) Representative image of immunofluorescence of neurons in microfluidic chambers labeled with βIII-tubulin (cyan), Rab11a (green), and f-Ctb555 (red). Scale bar, 100 µm. (C) Representative image of neurons in the cell body compartment, microgrooves, and distal axons. Scale bar, 10 µm.
- **Labeling signaling endosomes with biotinylated BDNF**
  1. Conjugation of biotinylated BDNF (BDNF-biotin) with fluorescently labeled (DyLight488) streptavidin (f-streptavidin488)
    We have previously published a protocol to monobiotinylate recombinant BDNF and to conjugate it to f-streptavidin (Stuardo et al., 2020). Here, we describe the protocol to conjugate commercially available BDNF-biotin as performed in Moya-Alvarado et al. (2023); both protocols were used in this publication.
    - Dissolve 5 µg of BDNF-biotin in 50 µL of neurobasal media in 0.1% BSA.
*Note: Prepare 2 µL aliquots so you do not freeze and defrost the BDNF-biotin more than once (store aliquots at -80 °C). BDNF-biotin is not stable when it is stored more diluted. The concentration of BDNF will be 100 µg/mL. According to the manufacturer’s instructions, BDNF can have one or two biotin molecules.*
    - Resuspend 10 µg of Streptavidin DyLight 488 in 100 µL of neurobasal medium with 0.1% BSA.
*Note: If f-streptavidin contains sodium azide, it will have to be dialyzed with mini dialysis devices.*
    - Add to an Eppendorf tube 2 µL of BDNF-biotin solution (100 μg/mL), 6 µL of f-strepavidin 488 (100 μg/mL), and 72 µL of neurobasal medium (supplemented with 0.1% BSA); mix up and down with a p200 pipette.
    - Incubate the solution for 20 min at 37 °C.
    - Then, dilute the mixture in 320 µL of neurobasal medium with 0.1% BSA at 37 °C to obtain a final solution of ~5 nM BDNF-biotin conjugated to f-streptavidin488 (or 150 ng/mL).
*Note: As a control for conjugated BDNF treatment, nonconjugated f-streptavidin488 at the same final concentration is used.*
  2. Multilabeling neuronal endosomes with fluorescent BDNF (f-BDNF), f-Ctb647 from axons, and fluorescent labeled Alexa Fluor 555 transferrin (f-transferrin 555) from cell bodies
    - Compartmentalized cortical neurons are grown as indicated in Section B until DIV 8.
    - Remove the media from the chamber (80 µL from each hole of the cell body compartment and 40 µL from each hole of the axonal compartment).
    - Add 80 µL of TrkB-fc (100 µg/mL) in neurobasal medium to each hole of the cell body compartment and 40 µL of neurobasal medium to each hole of the axonal compartment for 60 min at 37 °C. This treatment neutralizes endogenous BDNF in the cell body compartment.
    - Then, remove the media from the axonal compartment and add f-Ctb647 (1 µg/mL) and f-BDNF488 (150 ng/mL) diluted in a total of 80 µL of neurobasal medium (1% BSA) for 180 min at 37 °C.
    - After 150 min of the abovementioned incubation, remove the media (80 µL from each hole) from the cell body compartment and then add 80 µL of neurobasal medium containing f-transferrin 555 (100 µg/mL) to each hole of the cell body compartment for the next 30 min to label early/recycling endosomes.
    - After 180 min, replace the medium in both compartments with warm PBS.
    - Then, proceed as indicated in Section D.
  3. Confocal microscopy settings for Figures 5 and 6
    - We used a Leica SP8 confocal microscope with LAS X version 3.5.5.19976 software. Laser Diode 405, Diode 638, OPSL 488, and OPSL 552.
    - Identify the cells using the EPI fluorescence system by Hoechst or Ctb555 staining in the cell body compartment using the 63× objective.
*Note: To visualize the cell body of neurons, we applied a 5× zoom. No zoom was applied for imaging axons in the axonal compartment or in the microgrooves.*
    - Take 6–14 pictures using an optical slice 1 µm (or less) thick using the Z-scan option.
*Note: In Figures 5 and 6, pictures of 1,024 × 1,024 pixel resolution were taken. In Figure 6, the following settings were applied: a gain of 850 and an offset O, with a 2%–5% intensity of 405 laser, 3%–10% 488 laser, 0.1%–0.5% 555 laser, and 0.1%–0.5% 647 laser. The setting conditions will vary depending on the experiment and must be set up for each sample.*
  4. Co-localization analysis using the fluorescence intensity plot profile as shown in Figure 5
    - Open ImageJ in the computer and load the image acquired by the confocal microscope.
    - Select from the Z-stack images the picture of the optical slice with higher fluorescence intensity from the channel labeling f-BDNF in Figure 5.
    - Select the option *Straight line* in ImageJ. Draw a line of 2–4 µm on top of the particle for analysis.
    - Add the line to the ROI (region of interest) manager to save the position of the line.
    - Open the option *Analyze* and select *Plot profile* for each channel.
*Note: We also performed an analysis of the co-localization of f-BDNF endosomes with other labels, such as phospho TrkB antibodies, to detect signaling receptors using single organelle analysis as described in the methods of Moya-Alvarado et al. (2023).*

## References

[1] Andres-Alonso M., Ammar M. R., Butnaru I., Gomes G. M., Acuña Sanhueza G., Raman R., Yuanxiang P., Borgmeyer M., Lopez-Rojas J., Raza S. A., .(2019). SIPA1L2 controls trafficking and local signaling of TrkB-containing amphisomes at presynaptic terminals. Nat. Commun. 10(1): e5448. 10.1038/s41467-019-13224-z PMC6884526 31784514

[2] Campenot R. B.(1977). Local control of neurite development by nerve growth factor. Proc. Natl. Acad. Sci. U.S.A. 74(10): 4516-4519. 270699 10.1073/pnas.74.10.4516 PMC431975

[3] De Vitis E., La Pesa V., Gervaso F., Romano A., Quattrini A., Gigli G., Moroni L. and Polini A.(2021). A microfabricated multi-compartment device for neuron and Schwann cell differentiation. Sci. Rep. 11(1): 7019. 33782434 10.1038/s41598-021-86300-4 PMC8007719

[4] Gluska S., Chein M., Rotem N., Ionescu A. and Perlson E.(2016). Tracking Quantum-Dot labeled neurotropic factors transport along primary neuronal axons in compartmental microfluidic chambers. Methods Cell Biol.: 365–387. 10.1016/bs.mcb.2015.06.016 26794524

[5] Guedes-Dias P. and Holzbaur E. L. F.(2019). Axonal transport: Driving synaptic function. Science 366(6462): eaaw9997. 31601744 10.1126/science.aaw9997 PMC6996143

[6] Hafner A. S., Donlin-Asp P. G., Leitch B., Herzog E. and Schuman E. M.(2019). Local protein synthesis is a ubiquitous feature of neuronal pre- and postsynaptic compartments. Science 364(6441): eaau3644. 31097639 10.1126/science.aau3644

[7] Hirokawa N., Niwa S. and Tanaka Y.(2010). Molecular Motors in Neurons: Transport Mechanisms and Roles in Brain Function, Development, and Disease. Neuron 68(4): 610-638. 21092854 10.1016/j.neuron.2010.09.039

[8] Jadhav A. D., Wei L. and Shi P.(2016). Compartmentalized Platforms for Neuro-Pharmacological Research. Curr Neuropharmacol 14(1): 72-86. 26813122 10.2174/1570159X13666150516000957 PMC4787287

[9] Kaech S. and Banker G.(2006). Culturing hippocampal neurons. Nat Protoc 1(5): 2406-2415. 17406484 10.1038/nprot.2006.356

[10] Koley S., Rozenbaum M., Fainzilber M. and Terenzio M.(2019). Translating regeneration: Local protein synthesis in the neuronal injury response. Neurosci. Res. 139: 26-36. 30321567 10.1016/j.neures.2018.10.003

[11] Lazo O. M. and Schiavo G.(2023). Rab10 regulates the sorting of internalised TrkB for retrograde axonal transport. eLife 12: e81532. 36897066 10.7554/eLife.81532 PMC10005780

[12] MacInnis B. L. and Campenot R. B.(2002). Retrograde Support of Neuronal Survival Without Retrograde Transport of Nerve Growth Factor. Science 295(5559): 1536-1539. 11799202 10.1126/science.1064913

[13] Moya-Alvarado G., Guerra M. V., Tiburcio R., Bravo E. and Bronfman F. C.(2022). The Rab11-regulated endocytic pathway and BDNF/TrkB signaling: Roles in plasticity changes and neurodegenerative diseases. Neurobiol. Dis. 171: 105796. 35728773 10.1016/j.nbd.2022.105796

[14] Moya-Alvarado G., Tiburcio-Felix R., Ibáñez M. R., Aguirre-Soto A. A., Guerra M. V., Wu C., Mobley W. C., Perlson E. and Bronfman F. C.(2023). BDNF/TrkB signaling endosomes in axons coordinate CREB/mTOR activation and protein synthesis in the cell body to induce dendritic growth in cortical neurons. eLife 12: e77455. 36826992 10.7554/eLife.77455 PMC9977295

[15] Panayotis N., Karpova A., Kreutz M. R. and Fainzilber M.(2015). Macromolecular transport in synapse to nucleus communication. Trends Neurosci. 38(2): 108-116. 25534890 10.1016/j.tins.2014.12.001

[16] Park J. W., Vahidi B., Taylor A. M., Rhee S. W. and Jeon N. L.(2006). Microfluidic culture platform for neuroscience research. Nat. Protoc. 1(4): 2128-2136. 17487204 10.1038/nprot.2006.316

[17] Pathak A., Clark S., Bronfman F. C., Deppmann C. D. and Carter B. D.(2020). Long‐distance regressive signaling in neural development and disease. WIREs Dev. Biol. 10(2): e382. 10.1002/wdev.382 PMC7655682 32391977

[18] Shigeoka T., Jung H., Jung J., Turner-Bridger B., Ohk J., Lin J. Q., Amieux P. S. and Holt C. E.(2016). Dynamic Axonal Translation in Developing and Mature Visual Circuits. Cell 166(1): 181-192. 27321671 10.1016/j.cell.2016.05.029 PMC4930487

[19] Southam K. A., King A. E., Blizzard C. A., McCormack G. H. and Dickson T. C.(2013). Microfluidic primary culture model of the lower motor neuron–neuromuscular junction circuit. J. Neurosci. Methods 218(2): 164-169. 23774648 10.1016/j.jneumeth.2013.06.002

[20] Stuardo N., Moya-Alvarado G., Ramírez C., Schiavo G. and Bronfman F. C.(2020). An Improved Protocol to Purify and Directly Mono-Biotinylate Recombinant BDNF in a Tube for Cellular Trafficking Studies in Neurons. J. Vis. Exp.: e3791/61262. 10.3791/61262 32716376

[21] Villarin J. M., McCurdy E. P., Martínez J. C. and Hengst U.(2016). Local synthesis of dynein cofactors matches retrograde transport to acutely changing demands. Nat. Commun. 7: 13865. 28000671 10.1038/ncomms13865 PMC5187584

[22] Wang T., Martin S., Nguyen T. H., Harper C. B., Gormal R. S., Martínez-Mármol R., Karunanithi S., Coulson E. J., Glass N. R., Cooper-White J. J., .(2016). Flux of signalling endosomes undergoing axonal retrograde transport is encoded by presynaptic activity and TrkB. Nat. Commun. 7: 12976. 27687129 10.1038/ncomms12976 PMC5427517

[23] Yamashita N. and Kuruvilla R.(2016). Neurotrophin signaling endosomes: biogenesis, regulation, and functions. Curr. Opin. Neurobiol. 39: 139-145. 27327126 10.1016/j.conb.2016.06.004 PMC4987223

[24] Zhang J., Yang H., Wu J., Zhang D., Wang Y. and Zhai J.(2022). Recent progresses in novel in vitro models of primary neurons: A biomaterial perspective. Front. Bioeng. Biotechnol. 10: e953031. 10.3389/fbioe.2022.953031 PMC9428288 36061442

